# Genetic Architecture of Flowering Time Differs Between Populations With Contrasting Demographic and Selective Histories

**DOI:** 10.1093/molbev/msad185

**Published:** 2023-08-21

**Authors:** Célia Neto, Angela Hancock

**Affiliations:** Molecular Basis of Adaptation Research Group, Max Planck Institute for Plant Breeding Research, Cologne, Germany; Molecular Basis of Adaptation Research Group, Max Planck Institute for Plant Breeding Research, Cologne, Germany

**Keywords:** directional selection, flowering time, complex traits, Fisher–Orr geometric model, adaptive walk‌

## Abstract

Understanding the evolutionary factors that impact the genetic architecture of traits is a central goal of evolutionary genetics. Here, we investigate how quantitative trait variation accumulated over time in populations that colonized a novel environment. We compare the genetic architecture of flowering time in *Arabidopsis* populations from the drought-prone Cape Verde Islands and their closest outgroup population from North Africa. We find that trait polygenicity is severely reduced in the island populations compared to the continental North African population. Further, trait architectures and reconstructed allelic histories best fit a model of strong directional selection in the islands in accord with a Fisher–Orr adaptive walk. Consistent with this, we find that large-effect variants that disrupt major flowering time genes (*FRI* and *FLC)* arose first, followed by smaller effect variants, including *ATX2* L125F, which is associated with a 4-day reduction in flowering time. The most recently arising flowering time-associated loci are not known to be directly involved in flowering time, consistent with an omnigenic signature developing as the population approaches its trait optimum. Surprisingly, we find no effect in the natural population of *EDI-Cvi-0* (*CRY2* V367M), an allele for which an effect was previously validated by introgression into a Eurasian line. Instead, our results suggest the previously observed effect of the *EDI-Cvi-0* allele on flowering time likely depends on genetic background, due to an epistatic interaction. Altogether, our results provide an empirical example of the effects demographic history and selection has on trait architecture.

## Introduction

Understanding how phenotypic variation is generated and maintained within populations and how this variation is encoded in the genome are central goals of evolutionary genetics (Barton and Keightley 2002; Lee et al. 2014; Josephs et al. 2017). Since Darwin and Wallace and the origin of evolutionary theory, there has been a debate over the relative importance of jumps and leaps versus gradual change in trait evolution (Wallace 1855; Darwin 1859; Gould and Eldredge 1972; Provine 2001). Developing a quantitative understanding of trait architectures and what shapes them is important in both fundamental and applied research. Specific parameters of interest include the number of variants that contribute to observed variation in a trait, the frequencies of those variants in the natural population, and their distributions of effect sizes. Estimates of these parameters in natural populations are needed to parameterize theoretical models, predict evolutionary outcomes under future scenarios, and design powerful trait-mapping studies. However, our understanding of the range of genetic architectures in nature and the factors that influence them is still remarkably limited.

Theory predicts that several parameters are likely to shape genetic architectures of quantitative traits in natural populations. These include the amount of heritable genetic variation available within a population, which is related to the long-term effective population size (N_e_), or the inverse of the time depth of the population, as well as the selective force and strength of selection acting on the trait (Gillespie 1983b; Orr 1998; Silander et al. 2007; Gomulkiewicz and Houle 2009; Gomulkiewicz et al. 2010; Barton et al. 2017; Sella and Barton 2019; Charlesworth et al. 2022). In large-N_e_ populations, if the trait of interest is evolving under stabilizing selection, new mutations with substantial effects on fitness-associated traits are likely to be removed rapidly by purifying selection. Variants found segregating in these large-N_e_ populations are therefore expected to be at low frequency, and any specific mutation should have only a minor impact on a trait. Conversely, in a population that experiences strong directional selection, beneficial large-effect variants may provide the first steps toward adaptation (Orr 1998, 2005).

Fisher's “infinitesimal model” assumes a population of infinite size evolving under stabilizing selection (Fisher 1918). As new mutations arise, they are expected to result in minute effects across the genome that pull individuals away from the fitness optimum and contribute to trait variation within the population. Fisher extended his infinitesimal model to include adaptation in a “geometric model” (Fisher 1930). Under this model, a population far from a high-dimensional phenotypic optimum moves closer to it using random mutations that arise over time. Fisher assumed that universal pleiotropy constrains evolution, providing further rationale for the idea that many variants of small phenotypic effect should underlie trait variation in the population (Fisher 1930). Recent work on human disease trait evolution, which is based on Fisher's infinitesimal and geometric models, shows support for these models. These studies examine the impact of variation arising in the context of a trait evolving under stabilizing selection (as is the case for many human disease traits) (Simons et al. 2018; Sella and Barton 2019) or after a weak or moderate shift in a selective pressure and thus a phenotype (Hayward and Sella 2022; Simons et al. 2022). However, the infinitesimal model is not sufficient to explain patterns that arise under very strong directional selection (Barton et al. 2017).

In the case where a very large environmental change occurs and available genetic variation cannot produce an adequate phenotypic shift, new large-effect loci are expected to play a prominent role in the adaptive process (Gillespie 1983b; Matuszewski et al. 2014; Orr 1998, 2002). Building on Fisher's model and subsequent theoretical advances (Kimura 1983; Gillespie 1983a; Kauffman and Levin 1987), Orr formulated a model of the genetics of adaptation that approximates theoretical and empirical data (Orr 1998). The resulting Fisher–Orr model asserts that, after a sudden change in the environment (e.g., following a major environmental perturbation or after colonization of a new habitat), a mutation-limited population will move toward the new fitness optimum through an “adaptive walk”. Under this model, the first steps of adaptation are likely to occur through few a large-effect mutations that overcome genetic drift to increase in frequency in the population (Orr 1998, 2002). Later in the adaptive walk, smaller effect mutations fine-tune phenotypic variation (Orr 2002). Overall, effect sizes are expected to follow an exponential distribution, with a few large impact variants and many small impact variants. This scenario can be contrasted with the expectation under stasis, where stabilizing selection mainly acts to preserve trait values that maximize fitness against newly arising deleterious variation. Under these conditions, the distribution of effect sizes is expected to be uniform, although it has been suggested that even under neutrality an exponential or similar distribution might be expected (Robertson 1967; Cotto and Day 2023). Experimental work on bacteria and yeast (Barrick et al. 2009; Frenkel et al. 2014; Good et al. 2017) has provided some empirical insights on how populations adapt over time as populations evolve. However, real populations may be more complex than these models assume. For example, real populations may be spatially structured and/or subject to spatial and temporal variation in selection pressures (Rausher and Delph 2015; Dittmar et al. 2016; Connallon and Hodgins 2021).

While theory provides expectations about how demographic and selective histories should impact trait architecture, natural systems that allow us to make direct comparisons are rare. For this, we need to study populations with known, contrasting histories. Here, we compare the genetic architecture of flowering time in *Arabidopsis* populations that colonized the Cape Verde Islands (CVI) with that of their closest outgroup population from Morocco.

**Fig. 1.**
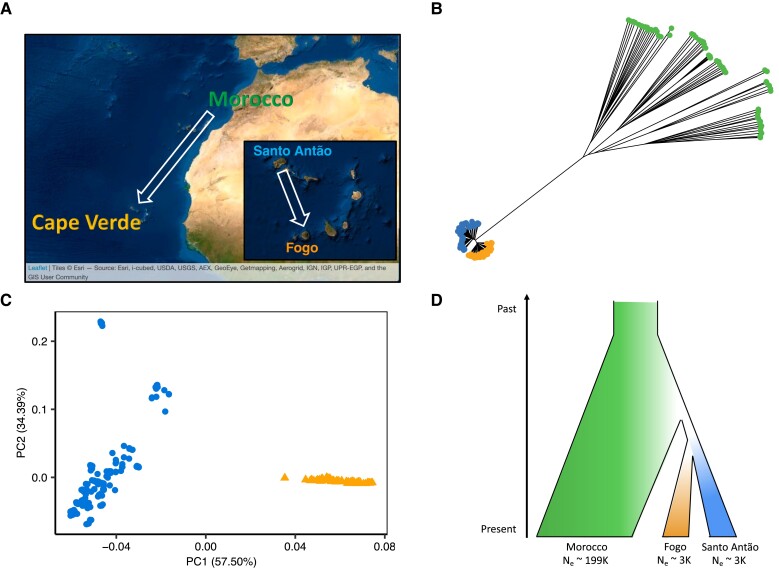
Geographical location and population history of CVI Arabidopsis. (*A*) Arabidopsis colonized Cape Verde 4–5 kya from North Africa. (*B*) Neighbor-joining tree showing the relationship between Moroccan and Cape Verdean individuals. (*C*) Principal component analysis (PCA) showing the differentiation between Santo Antão (circles) and Fogo individuals (triangles). (*D*) Schematic of Cape Verde Arabidopsis history.

The colonization history of *Arabidopsis thaliana* in the CVI is well-defined (Fulgione et al. 2022), providing an effective case for investigating how population history and selection impact trait architecture. The CVI lies at the geographic and climatic edge of the species distribution. Natural populations of *A. thaliana* populate two CVI: Santo Antão and Fogo, where they are restricted to altitudes greater than 900 m. The islands were colonized approximately 4–5 kya by long-range dispersal from a North African progenitor (fig. 1), best represented today by the Moroccan population (Fulgione et al. 2022). Consistent with colonization through an extreme bottleneck, diversity is strongly reduced in CVI relative to Morocco (Durvasula et al. 2017; Fulgione et al. 2022). Estimates of long-term N_e_ based on diversity (θ_W_) and estimated mutation rate (Ossowski et al. 2010; Exposito-Alonso et al. 2018; Wang et al. 2019; Weng et al. 2019; Fulgione et al. 2022) are approximately 60 to 90 times higher in the Moroccan population compared to Santo Antão and Fogo, respectively (N_e Santo Antão_ = 2–4 K, N_e__Fogo_ = 3–5 K, N_e Morocco_ = 176–323 K, for a mutation rate between 4.3 × 10^−9^ and 7.9 × 10^−9^). The near-complete bottlenecks during colonization eliminated 99.9% of the shared variation with the continent, producing phylogenetically distinct variation-depauperate populations in each of the two islands (Fulgione et al. 2022) in which it is straightforward to assign ancestral states to variants. This case provides the possibility to directly compare trait evolution in the long-established, high-N_e_ Moroccan population, with a population coalescence time of approximately 1 mya (Durvasula et al. 2017), and two distinct recently established Cape Verde island populations, where variation is expected to have arisen de novo since colonization (Fulgione et al. 2022). This situation in some ways parallels experimental evolution studies, where it is possible to investigate how mutations accumulated over time in the population.

Reproductive timing is often a crucial element of an organism's life cycle and a primary fitness component in nature (Stearns 1992; Fabian and Flatt 2012). In plants, flowering time is an excellent model trait for connecting ecological and evolutionary factors with their impacts on fitness. Flowering too early—before an individual has had time to accumulate sufficient resources—may limit reproductive capacity. Conversely, flowering too late can result in low or zero fitness in drought-prone environments (Ludlow 1989; McKay et al. 2003). Several studies have shown that flowering time is an important component of reproductive success (Hall and Willis 2006; Franks et al. 2007; Korves et al. 2007; Li et al. 2010; Anderson et al. 2011; Ågren and Schemske 2012; Dittmar et al. 2014; Ågren et al. 2017), a finding that is further supported by observed clinal patterns (Ducousso et al. 1996; Hurme et al. 2000; Olsson and Ågren 2002; Caicedo et al. 2004; Stinchcombe et al. 2004).

We previously showed that flowering time is reduced in CVI *Arabidopsis* populations, which increases reproductive success through a drought escape mechanism (Fulgione et al. 2022). We identified two large impact variants responsible for the convergent reduction in flowering time between the two CVI, Santo Antão and Fogo. These variants disrupted two interacting genes (*FRI* in one island and *FLC* in the other), reducing the flowering time by 27–31 days in each case (Fulgione et al. 2022). However, since flowering time is a complex trait, its basis is expected to be polygenic, with contributions from many loci genome-wide (Mouradov et al. 2002; Salomé et al. 2011; Andrés and Coupland 2012; Bouché et al. 2016; Zan and Carlborg 2018, 2019). In this mutation-limited population facing an abrupt environmental change, we expect adaptation to follow an adaptive walk toward the new optimum. According to theory, large-effect mutations should then arise early in the walk, followed by smaller ones later, with the total effect size distribution roughly fitting a negative exponential (Orr 1998).

Here, we examine the full genetic architecture of the time to first flowering in Cape Verde and compare it to the architecture in the Moroccan outgroup population. We find that polygenicity is severely reduced in the colonizing populations, consistent with the recent coalescent times of these populations. We identify loci associated with the reduction in the time to first flowering, hereafter flowering time, on the islands and show that these have smaller effects and arose more recently than the two large-effect loss-of-function mutations previously identified in CVI (*FRI* K232X and *FLC* R3X) (Fulgione et al. 2022). Overall, this approach allows us to examine how polygenic architecture built up over time in an ecologically relevant trait and provides an empirical example of the effects of demographic history and selection on trait architecture.

## Results

### Genome-Wide Association Studies Peaks Contain Loci Implicated in Core Flowering Time Pathways as Well as Peripheral Pathways

**Fig. 2.**
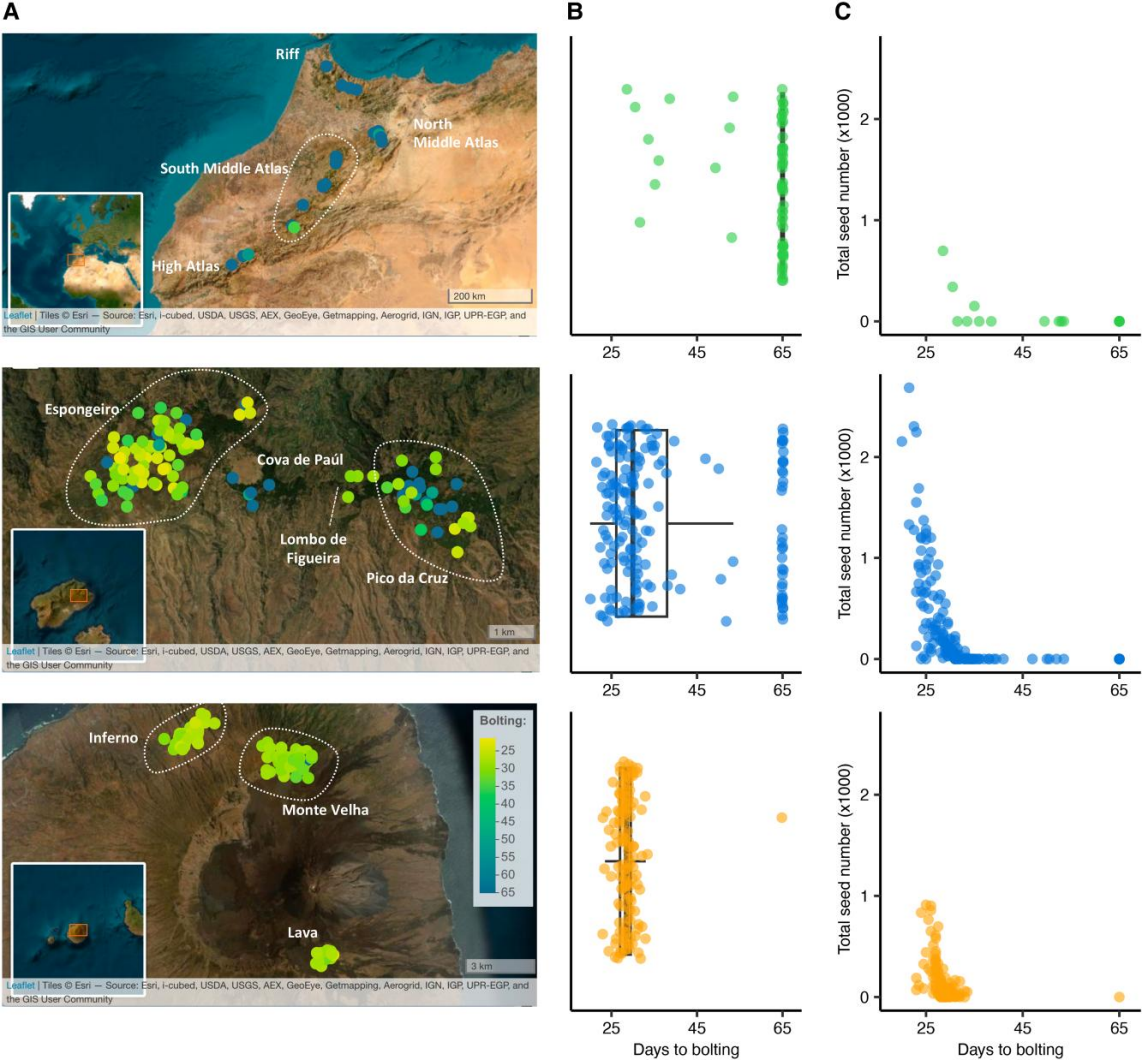
Flowering time varies in the populations and is correlated with fitness. Results are shown from top to bottom for Moroccan, Santo Antão, and Fogo populations, respectively. (*A*) Spatial distribution of variation in flowering time. (*B*) Phenotypic distribution of flowering time per population. Each dot corresponds to the median across four replicates per line. (*C*) Scatter plot showing the time to first flowering versus seed production, a proxy for fitness.

We propagated lines originally sampled from the two CVI and Morocco (fig. 2*a*) together in a growth chamber programed to simulate CVI field conditions, based on data we collected using data loggers placed in Cape Verde. These included light intensity, basic soil characteristics, daily precipitation, and hourly temperature and humidity. We measured the time it took for plants to bolt, hereafter referred to as “flowering time”. On average, flowering time was faster in Cape Verde compared to Morocco and inversely correlated with seed production in a simulated CVI environment (fig. 2*b*–*c*) (Fulgione et al. 2022). We calculated broad-sense heritability based on repeatability across replicates in an analysis of variance framework (line effect), as well as narrow-sense heritability based on the proportion of trait variance explained by relatedness (PVE) (Zhou et al. 2013). Heritabilities were high (table 1): The line effect ranged from 51% to 85% in a model that controlled for block effects and the PVE based on the trait correlation with the relatedness matrix was greater than 95%, implying that a large proportion of the variation in flowering time is genetic.

**Table 1.** Heritability of Flowering Time Under CVI Simulated Conditions and the Inferred Number of Loci Contributing to Trait Variation Using Three Complementary Approaches.

	Heritability		Number of loci		
	H^2^ = $\frac{var_{line}}{var_{line}+var_{residual}}$^a^	PVE^b^ (95%CI)^c^	BSLMM^d^	Clump	LocalScore
Morocco	80.10	99.96 (99.8–99.99)	39	6666	353
Santo Antão	85.28	98.3 (97.6–98.8)	10	104	6
Fogo	51.10	95.2 (92.5–97.6)	3	159	26

a Broad-sense heritability calculated across replicates (in %).

b Percentage of phenotypic variance explained by relatedness (narrow-sense heritability, in %).

c 95% CI across ten runs in BSLMM (in %).

d Bayesian sparse LMM.

To identify the genetic loci underlying flowering time variation, we conducted genome-wide association studies (GWAS) separately for each population using a linear mixed model (LMM) approach that controls for relatedness using a kinship matrix (Zhou and Stephens 2012) (supplementary fig. S1, Supplementary Material online). Since we already had clear functional evidence that two variants, *FRI* K232X in Santo Antão and *FLC* R3X in Fogo, strongly affect flowering time in CVI (Fulgione et al. 2022), we accounted for these in subsequent analyses. In particular, since *FRI* K232X segregates in Santo Antão, we included this variant as a covariate in the LMM. To reduce redundancy resulting from local linkage disequilibrium (LD) and to increase the power to detect associations across haplotypes, we used an approach that aggregates association signals based on LD between markers within a genomic region (Fariello et al. 2017; Bonhomme et al. 2019).

In this method, *P*-values from GWAS are integrated across haplotypes along genomic regions based on LD (Mercier and Daudin 2001; Fariello et al. 2017; Bonhomme et al. 2019). These are converted to “local scores”, such that strongly associated regions have the most positive scores. In effect, this method identifies variants that represent the signal in larger haplotypes. The local score approach is in some ways analogous to window-based identification of candidate loci, but it has been shown to have higher power than these approaches (Fariello et al. 2017).

**Fig. 3.**
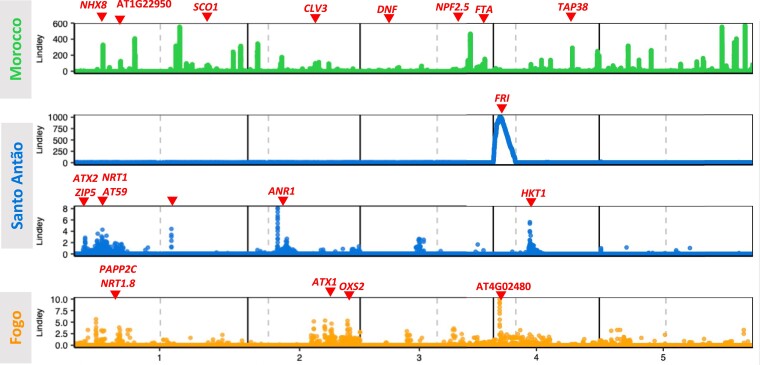
GWAS for flowering time. Manhattan plots for the three populations (Morocco, Santo Antão, and Fogo) show associations with flowering time under CVI simulated conditions. The chromosome position is shown on the x-axis and the Lindley score from the local score approach is shown on the y-axis. Candidate genes are denoted by arrows. For Santo Antão, the top panel shows GWAS results with all genotyped markers and the bottom panel shows GWAS results with *FRI* K232*X* as a covariate.

In Santo Antão, we identified five significantly associated regions (fig. 3), containing variants in seven genic candidate loci. Among these were several genes with known direct links to flowering time pathways (*NRT1*, *AT59*, *ANR1*, *ATX2*, and *HKT1*) as well as genes involved in zinc transport and leaf and stem morphogenesis (*ZIP5* and *ANAC036*). An especially strong candidate is *ATX2*, an H3K4-specific methyltransferase that affects flowering time in a *FRI*-dependent manner by regulating levels of FLC mRNA (Pien et al. 2008; Saleh et al. 2008; Shafiq et al. 2014). In the natural population, we identified a missense variant in this gene, L125F, segregating at 66.67% frequency and associated with a 4-day reduction in flowering time in the *FRI* derived background (Tukey test *FRI*-*ATX2*, Der-Der/Der-Anc: diff = −5.43 days, *P*-value = 4.82 × 10^−10^; beta from genome-wide efficient mixedmodel association (GEMMA) LMM with *FRI* as a covariate = −3.8 days, *P* = 0.0089). Our results agree with previous mutant analyses showing a strong effect of *ATX2* on the number of leaves at bolting (as a proxy for flowering time) (Shafiq et al. 2014). Overall, in Santo Antão, we found that candidate loci comprise genes involved in core flowering pathways as well as those with less direct links to flowering time.

Previous studies that used mapping populations derived from a cross between the *A. thaliana* lines Cvi-0 (derived from a plant from Santo Antão) (Fulgione et al. 2022) and Ler-0 (derived from a Eurasian plant) (Alonso-Blanco, Peeters, et al. 1998; Alcázar et al. 2014) identified a CVI haplotype that resulted in daylength insensitive early flowering (Alonso-Blanco, El-Assal, et al. 1998). This haplotype was initially designated the *EDI-Cvi-0* (*Early Day-length Insensitive*) allele and was later localized to a specific missense variant (V367M) in the Cryptochrome 2 blue light photoreceptor, *CRY2* (Alonso-Blanco, Peeters, et al. 1998; El-Din El-Assal et al. 2001; Keurentjes et al. 2007). When introgressed into a European background (Ler-0), *EDI-Cvi-0* has a large impact on flowering time, reducing the time to flower such that leaf number at flowering is 18–22 leaves less in short day conditions (Alonso-Blanco, El-Assal, et al. 1998). Subsequently, using transgenic allelic exchange, it was shown that the Cvi-0 specific V367 variant was sufficient to explain the difference in flowering time between the Cvi-0 allele relative to Ler-0 (El-Din El-Assal et al. 2001). This represents one of the earliest and best-validated cases of a natural variant's functional impact on trait variation.

We specifically examined the *CRY2* 367M allele in the natural population, which we found was private to and segregating at high frequency in Santo Antão (90%). In GWAS, we found neither a significant nor a suggestive effect of this variant in the natural population (LMM in GWAS, *P*-value = 0.945; fig. 3, supplementary figs. S1 and S2, Supplementary Material online), even after correction for the effect of *FRI* 232X (LMM, *P*-value = 0.318). The discrepancy between our results and previous findings could have been due to a difference in the conditions in our experiment. To test this hypothesis, we grew the near-isogenic lines (NILs) that were previously used to map the *EDI-Cvi-0* allele (Alonso-Blanco, El-Assal, et al. 1998; Keurentjes et al. 2007) in our growth conditions. These NILs contain the *EDI-Cvi-0* (*CRY2*) locus introgressed into the European (Ler-0) background (El-Din El-Assal et al. 2001; Keurentjes et al. 2007). This experiment recapitulated the previously reported reduction in flowering time due to *EDI-Cvi-0*, showing that in the European genetic background, even under simulated CVI conditions, *CRY2* 367M reduces flowering time (supplementary fig. S2, Supplementary Material online) (Alonso-Blanco, Peeters, et al. 1998; El-Din El-Assal et al. 2001). When adding *CRY2* to a phenotype-prediction model alongside the other associated variants identified here, we found no evidence for a marginal or interaction effect. An epistatic effect due to a variant that is fixed in Santo Antão relative to Ler-0 is still a possibility. Another possibility is that a variant not carried by Cvi-0 but in LD with *CRY2* 367M that increases flowering time interferes with the detection of *CRY2* V367M in GWAS. To investigate this, we identified variants in high LD with *CRY2* V367M in Santo Antão. Of the ten variants with *r*^2^ > 0.8 with *CRY2* V367M, we found no clear flowering time candidate loci represented. Still, we cannot rule out the possibility that variants in LD with *CRY2* V367M in the natural population could affect our power to detect *CRY2* V367M in GWAS. Further work will be needed to understand the role of the *CRY2* variant on trait variation in Santo Antão and to determine with certainty whether the lack of signal there is due to a difference in the genetic background between Cvi-0 and Ler-0.

Next, we conducted GWAS in the Fogo population. Previously, we showed that the reduction in flowering time in Fogo is largely due to a fixed major effect mutation in the core vernalization pathway (*FLC* 3X) (Fulgione et al. 2022). In this study, GWAS revealed several additional smaller-effect associations with flowering time. We found signals of association near *PAPP2C* (AT1G22280), the gene that encodes a phosphatase that interacts with phytochromes A and B (Phee et al. 2008), and *OXS2* (AT2G41900), which encodes a stress-induced transcription factor that interacts with SOC1, FT (the gene symbol for FLOWERING TIME LOCUS T), and FD (the gene symbol for FLOWERING LOCUS D; three proteins known to induce the transition to flowering) (Blanvillain et al. 2011). The strongest association signal in Fogo overlapped AT4G02480, which encodes an AAA-type ATPase protein. Although its specific function is unknown, AT4G02480 has been shown to interact with the core flowering time proteins FKF (the gene symbol for FLAVIN-BINDING, KELCH REPEAT, F BOX 1;) and GI (the gene symbol for GIGANTEA), which regulate CO (the gene symbol for CONSTANS) protein stability for photoperiod control of flowering (Song et al. 2014). We also identified associations at two other candidate genes belonging to gene families associated with flowering time in Santo Antão: *ATX1* (AT2G31650) and *NPF5.15* (AT1G22570). ATX1 is a histone-lysine *N*-methyltransferase involved in the formation, placement, and identity of floral organs, and the epigenetic control of *FLC* (Pien et al. 2008). *ATX1* is also involved in seed germination, stomatal aperture, water loss, and sensitivity to dehydration stress (Ding et al. 2011, p. 1). In NPF5.15, a protein involved in nitrate and hormone transport (Léran et al. 2014), a nonsense variant is associated with flowering time in Fogo.

In the Moroccan GWAS, a large number of genomic loci were implicated in flowering time (fig. 3). These include nonsynonymous variants in genes directly involved in flowering time as well as loci involved in other traits. Loci directly implicated in flowering time include AT3G19140 (*DNF* L7V), AT2G27250 (*CLV3* M1fs), and AT1G22950 (S131T/G). *DNF* is an E3 ligase that represses *CO* and is crucial to distinguish between long and short days, preventing flowering in short days (Morris et al. 2010; Morris and Jackson 2010). *CLV3* is one of the three *CLAVATA* genes controlling the size of the shoot apical meristem in *Arabidopsis* and it regulates shoot and floral meristem development. AT1G22950 participates in the epigenetic repression of several members of the MADS-box transcription factor family during vegetative development via histone modification. It directly targets *FLC* and mutants disrupt the cold-induced Polycomb-mediated silencing underlying vernalization (Bloomer and Dean 2017).

Loci with GWAS signals that are not directly linked to the flowering-time pathway may indirectly contribute to variation in flowering time. In Morocco, we identified signals for variants in genes involved in mineral uptake and processing (*NPF2.2*, *NPF2.5*, and *NHX8*), photosynthesis and growth (*TAP38* and *SCO1*), and response to ultraviolet radiation and drought stress (*MPK1* and *FTA*). Overall, GWAS results from the three populations included a mix of loci involved directly in flowering time and those involved in other linked pathways.

### The Genetic Architecture of Flowering Time is Less Polygenic in the CVI

We next used the results from GWAS to assess whether trait polygenicity differed between the CVI and Morocco. We hypothesized that the flowering time trait would be less polygenic in the younger, less genetically diverse island populations compared to the older Moroccan populations. Since there is no single gold-standard method to infer trait architecture, we tested this hypothesis by applying three complementary approaches to estimate the number of loci contributing to the trait. These included an approach that estimates trait architecture assuming a mixture of large and small to infinitesimal effects (BSLMM) (Zhou et al. 2013), as well as two distinct approaches to reduce redundancy in GWAS results based on local LD. To estimate the genetic architecture from GWAS results by reducing redundancy due to local linkage (LD) in these, we applied 1) a clumping algorithm that maintains only the variants with *r*^2^ < 0.8 (Purcell et al. 2007) and 2) the “local score” approach described above (Fariello et al. 2017; Bonhomme et al. 2019).

The estimated numbers of loci contributing to the trait varied across methods, but all approaches resulted in the same pattern: many more loci underlie flowering time variation in the Moroccan population compared to the island populations (fig. 3, table 1). The estimated number of loci impacting the trait tended to be lowest with the BSLMM approach. This may be due to its integration of sparsity and shrinkage across the set of genome-wide associated loci in contrast to the other approaches that only remove local redundancy due to LD. The estimate from BSLMM may therefore be more conservative, but it is less likely to include causative loci than the other approaches. The local score method resulted in an intermediate number of loci compared to the extremes from the other two methods. Below, we use the local score results to investigate the evolutionary history of sets of trait-associated loci.

A limitation of this study was the relatively small sample size for GWAS in the Moroccan population (*n* = 62). Although we likely captured most genetic variation segregating at an appreciable frequency in CVI, we are underpowered in Morocco, where genetic variation is very high. However, the smaller sample size in Morocco should lead to an underestimate of the number of loci contributing to the trait in Morocco, so our finding that the flowering time trait is more polygenic in Morocco compared to the island populations should be robust to sample size differences.

### Patterns in the Cape Verde Populations are Consistent With Strong Directional Selection Under a Fisher–Orr Adaptive Walk Model

**Fig. 4.**
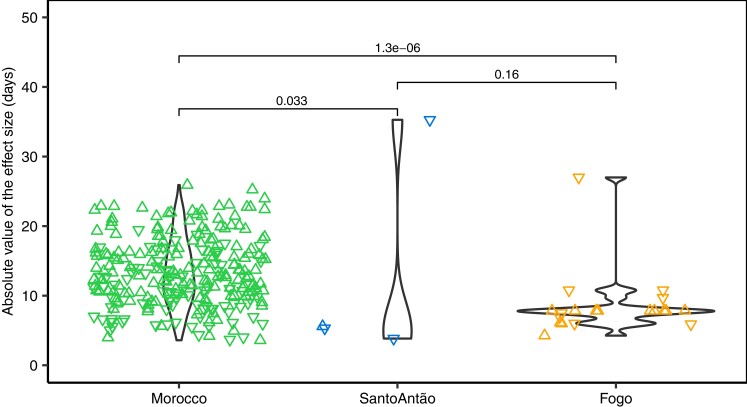
Effect size distributions of flowering time-associated loci. The absolute value of the effect size in days (y-axis) per population (x-axis). Each dot represents one SNP per candidate locus identified with the local score approach and with a *r*^2^< 0.5. Triangles pointed up represent variants with an estimated positive effect size, delaying flowering, while triangles pointed down represent variants with an estimated negative effect size, reducing flowering time. Although *FLC* R3*X* is fixed in Fogo, and not identified through GWAS, it is included here for completeness. *P*-values shown are from MW tests.

Under very strong directional selection, an exponential distribution of effect sizes is expected, with the largest-effect variants arising early in the adaptive walk (Orr 1998, 2005). In contrast, under a model of stabilizing selection or weak directional selection, many loci with a uniform distribution of effect sizes are expected to contribute to trait variation (Orr 1998; Barton et al. 2017; Koch and Sunyaev 2021; Simons et al. 2022). We investigated the distribution of effect sizes (ß estimated in GEMMA with an LMM) in each population using one representative variant per candidate locus (identified with the local score approach and LD-pruned to remove SNPs with *r*^2^ > 0.5) (fig. 4, supplementary table S1, Supplementary Material online). We manually added information about *FLC* R3X, which is fixed in Fogo. First, we compared the effect size distributions for the three populations and found that the Moroccan distribution is significantly different from the two islands’ [Mann–Whitney (MW) test, Morocco–Santo Antão: *P*-value = 0.033, Morocco–Fogo: *P*-value = 1.3 × 10^−6^). Then, to investigate whether these effect sizes fit a uniform distribution, as predicted under Fisher's infinitesimal model (Fisher 1918), or an exponential distribution, as predicted by the Fisher–Orr geometric model of adaptation (Fisher 1930; Orr 1998), we compared the fit of these two classes of distributions to the effect size distribution per population using a maximum likelihood approach. In Morocco, the distribution of effect sizes best fit a uniform distribution (Aikake Information Criterion [AIC]: exponential: 1810.13, uniform: 1568.97), while in Santo Antão and Fogo, effect size distributions best fit an exponential distribution (AIC _Santo Antão_: exponential: 30.21, uniform: 31.58; AIC _Fogo_: exponential: 127.94, uniform: 128.96). Overall, our results are consistent with evolution dominated by stabilizing selection in the Moroccan population and with strong directional selection in the CVI populations.

**Fig. 5.**
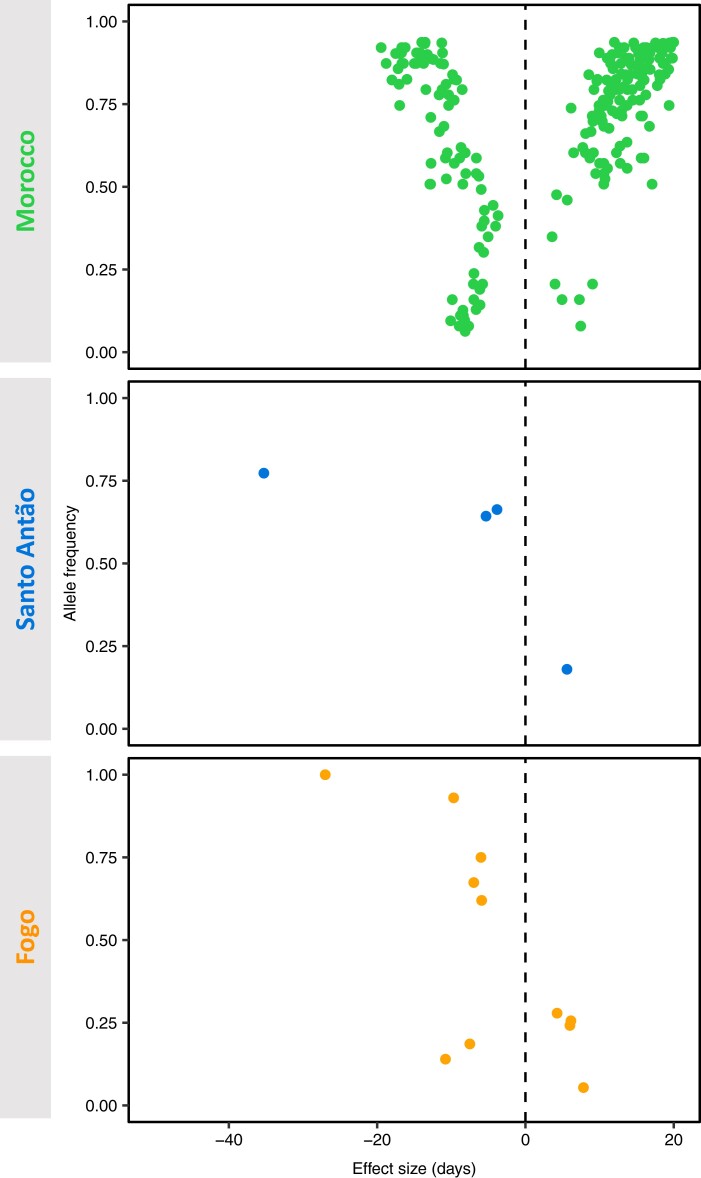
Relationship between allele frequency and effect size for flowering time-associated variants in the three populations. Effect size in days (x-axis) for *FRI* 232*X*, *FLC* 3*X*, and each SNP tagging a candidate locus (each dot) and its respective allele frequency on the population (y-axis). A negative effect size corresponds to early flowering and a positive effect size to late flowering.

We next investigated the relationship between allele frequencies and their associated effects to determine whether derived alleles tended to increase or decrease flowering time. In Morocco, we found that effect size was positively correlated with derived allele frequency (Pearson's *R* = 0.34, *P*-value = 1.98 × 10^−8^; fig. 5). When we specifically tested the effect for loci that increased versus decreased flowering time, we found that an increase in flowering time (i.e., later flowering) was significantly positively correlated with derived allele frequency (Pearson's *R* = 0.65, *P*-value < 2.2 × 10^−16^). On the other hand, for loci that decreased flowering time, we observed a negative correlation between flowering time and derived allele frequency (Pearson's *R* = −0.72, *P*-value = 1.44 × 10^−15^). On the islands, we found that alleles that accelerate flowering are mainly present at high frequencies while alleles that delay flowering are present at low frequencies, suggesting a history of directional selection. With so few variants, statistical analysis of correlations had low power, especially in Santo Antão, but the magnitudes of associations were strong in both cases (Santo Antão: Pearson's *R* = −0.74, *P*-value = 0.263, Fogo: Pearson's *R* = −0.76, *P*-value = 9.84 × 10^−5^; fig. 5). Our results are consistent with a predominant force of stabilizing selection acting to maintain late flowering in Morocco and parallel directional selection for earlier flowering in the islands. Consistent with results from fitness experiments (Fulgione et al. 2022), these results imply that in *Arabidopsis* populations that colonized Cape Verde, selection favored alleles that reduced flowering time because it allowed the populations to reproduce even with the shorter growing season. Conversely, in Morocco, where the growing season is longer, alleles that cause flowering to be later would tend to be beneficial.

**Fig. 6.**
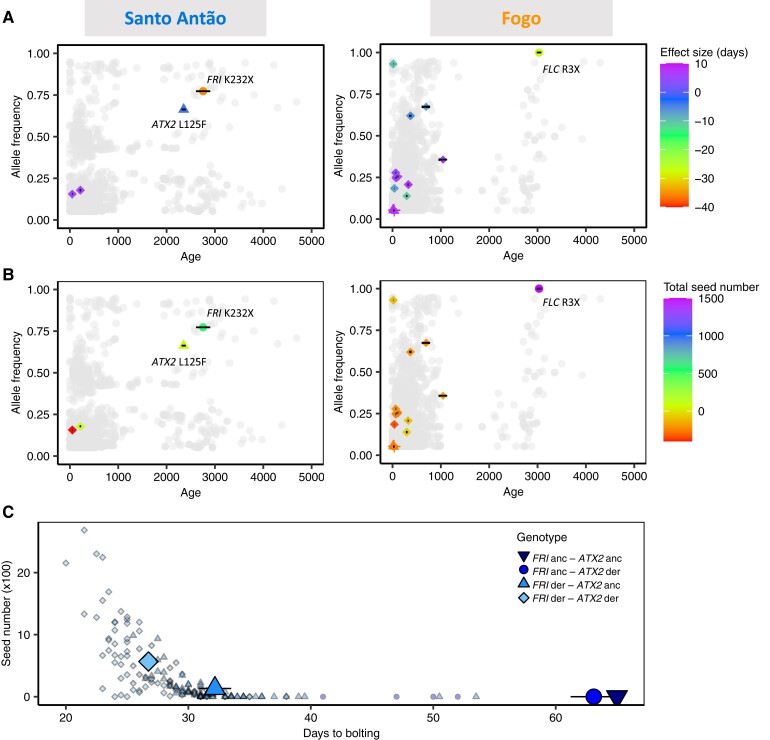
Relationship between age and allele frequency of loci implicated in flowering time and fitness in CVI. In (*A* and *B*), age estimates (in years) versus allele frequency for associated loci are shown in comparison to the genomic background. Colored SNPs represent associated variants, with colors matching their estimated effect sizes in days and seed number (*A* and *B*, respectively), and shape their predicted impacts (based on SnpEff annotation; circles are high impact, triangles moderate, diamonds are modifiers, and cross low effect variants). Each gray dot represents one SNP in an LD-pruned genome. The left panels refer to Santo Antão and the right panels to Fogo. Horizontal black lines on the associated variants represent 95% CI of estimated age. (*C*) Effects of allelic combinations between the two major candidates from Santo Antão, *FRI*, and *ATX2*, respectively, on time to flower (x-axis) and fitness (y-axis). Each small dot represents one line from the Santo Antão natural population, and large symbols the average per genotype category.

The near-complete loss of variation that occurred with the colonization of the two CVI provides a rare opportunity to examine how variation in a quantitative trait builds up over time. To this end, we next estimated the ages of loci identified in GWAS together with *FRI* 232X and *FLC* 3X using a coalescent approach (Speidel et al. 2019). On both islands, older flowering time-associated variants are at higher frequencies than younger variants (Santo Antão: Pearson's *R* = 0.999, *P*-value = 0.000461; Fogo: Pearson's *R* = 0.659, *P*-value = 0.0008517; fig. 6, supplementary table S2, Supplementary Material online). In Santo Antão, the oldest candidate variant, with an age estimated at approx. 2,700 years, causes a premature truncation of *FRI* K232X and was soon followed by a moderate effect missense mutation in *ATX2* (L125F, 2,300 years ago). Smaller effect loci, mainly modifiers, appeared more recently (in the last 200 years). In Fogo, *FLC* R3X arose approx. 3,000 years ago, followed by variants with smaller effects and moderate predicted functional effects in the last 1,000 years, with most arising in the last 500 years.

Overall, these results are consistent with a model where flowering time decreased on the archipelago through an adaptive walk, with large-effect size mutations arising first and quickly increasing in frequency (*FRI* K232X and *FLC* R3X), followed more recently by smaller effect size alleles. The most recently arising and lowest frequency loci often act to increase the time to flowering. These could be fine-tuning flowering time as expected under a Fisher–Orr model or they could be deleterious variants that have not yet been purged from the population.

## Discussion

Understanding trait architectures and the factors that shape them is a central goal in evolutionary biology, and it has applications in medical genetics, conservation genetics, and breeding (Falconer and Mackay 1996; Walsh and Lynch 2018; Bomblies and Peichel 2022; Charlesworth et al. 2022). Consistent with the infinitesimal model (Fisher 1918), highly polygenic architectures have been found in cases where population diversity is high and stabilizing selection or weak directional selection is the dominant evolutionary force acting on the population (Rockman 2012). For example, in *Drosophila*, body size variation has been attributed to hundreds to thousands of genomic regions (Turner et al. 2011; Pallares et al. 2023), and similarly, in mice, thousands of loci are needed to explain variation in body size (Reed et al. 2008). In a European human population, a study of more than 5 million individuals determined 12,111 associated SNPs could explain nearly all SNP heritability in height (Yengo et al. 2022). Expression QTL studies similarly tend to find that overall expression is highly polygenic (Josephs et al. 2015; He et al. 2016; Liu et al. 2019; Võsa et al. 2021). Overall, there is considerable evidence that traits in natural populations are often polygenic.

However, when selection is very strong and the population is mutation-limited with respect to the selected trait, large-effect variants may be important in adaptation, causing the infinitesimal model to break down (see Barton et al. 2017, p. 57). Extending on Fisher's geometric model, Orr produced a model of adaptation in which a mutation-limited population facing a sudden environmental shift follows an adaptive walk toward the new fitness optimum (Orr 1998, 2002). Empirical support for the Fisher–Orr geometric model comes from a broad range of traits and species (Dittmar et al. 2016; Connallon and Hodgins 2021). These include cases in microbial evolution (Arjan et al. 1999; Barrick et al. 2009; Schoustra et al. 2009; Szendro et al. 2013; Good et al. 2017); “industrial melanism” in *Biston betularia*, which is caused by a single large-effect locus that results in darker pigmentation and which rose to high frequency in highly industrial areas (Saccheri et al. 2008; van’t Hof et al. 2011, 2016); color patterning in mice living on pale coastal sand dunes versus dark lava or mainland environments, which is determined by variation in two genes, *Mc1r* and *Agouti* that together explain ∼40% of variation in pigmentation (Nachman et al. 2003; Hoekstra et al. 2006; Steiner et al. 2007; Weber et al. 2013); body shape variation between marine and freshwater three-spine stickleback fish, which is controlled by two large-effect loci and a few more small effect loci (Shapiro et al. 2004; Colosimo et al. 2005; Chan et al. 2010; Jones et al. 2012; Rogers et al. 2012; Peichel and Marques 2017; Schluter et al. 2021); and beak size variation in Darwin's finches reflecting dietary specialization, which is determined by up to six loci of large effect that explain up to 46% of the phenotypic variation (Lamichhaney et al. 2015; Enbody et al. 2022). In each of these examples, a population adapted to a distant optimum, using variants that had appreciable effects and explained a large proportion of the total phenotypic variance, similar to the island populations we studied here.

It is often implicitly assumed that trait architectures will be similar across populations. However, within a species, the demographic and selective histories of individual populations may vary, which can impact the genetic architectures of traits (Gomulkiewicz and Houle 2009). In this study, we asked whether the genetic architecture of a trait that contributes to adaptation in island-colonizing populations differs from that in the closest continental outgroup population. We compared the genetic architecture of flowering time in *Arabidopsis thaliana* populations that recently (ca. 4–5 kya) colonized a novel environment defined by a short growing season with their closest mainland outgroup. We hypothesized that strong directional selection in the Cape Verde populations combined with a relatively recent founder event would result in a pattern that is consistent with Orr's model of an adaptive walk (Orr 1998). Conversely, in the much older continental Moroccan population, which coalesces at approx. 1 mya, we hypothesized that the fitness optimum would largely have been reached and that patterns would be consistent with stabilizing selection or weak directional selection, as expected under an infinitesimal model of evolution (Fisher 1918; Barton et al. 2017).

We found several lines of evidence that the CVI populations were evolving under strong directional selection consistent with a Fisher–Orr geometric model. First, we found that polygenicity of the genetic architecture of the flowering time was reduced in the Cape Verde populations relative to the Moroccan population. Second, we found that effect sizes of trait-associated variants in the CVI populations were exponentially distributed, in contrast to the uniform distribution of effect sizes in Morocco. Although an exponential distribution of effects may be possible under neutral evolution (Robertson 1967; Cotto and Day 2023), other aspects of our results provide further evidence for strong directional selection. Third, based on reconstructed ages of the trait-associated variants, those implicated in large flowering time reductions arose first in CVI (*FLC* 3X, *FRI* 232X, and *ATX2* 125F) and smaller-effect variants arose more recently. Finally, the strong effect of reduced flowering time and the specific large-effect variants on fitness further support the role of these variants in adaptation. Taken together, the combination of an exponential distribution of effect sizes and strong fitness effects of *FLC* 3X in Fogo, and *FRI* 232X and *ATX2 125F* in Santo Antão provide evidence for an adaptive walk, consistent with a Fisher–Orr model.

Although we found evidence for an adaptive walk in the CVI populations, there are at least two ways in which the populations do not perfectly fit the Fisher–Orr model. First, *FRI* 232X alone and in combination with *ATX2* 125F has strong effects on flowering time and fitness (fig. 6), but neither variant is fixed in Santo Antão (supplementary fig. S3, Supplementary Material online). This could potentially be explained partly by population structure within the island, which likely developed early (Fulgione et al. 2022), and partially in response to a spatially variable climate. Specifically, the humid trade winds that provide much of the total moisture to the islands produce an east–west gradient in humidity during the wet season (Brochmann et al. 1997; Fulgione et al. 2022; Elfarargi et al. 2023). We are currently extremely limited in available models of quantitative traits, with existing models tending to assume random mating. Models that integrate population structure *and* that are realistic, that are informed by real data, will be important to advance our ability to make nuanced comparisons between models and data. Second, while there is strong evidence based on fitness in simulated CVI conditions (fig. 6) that the early arising variants were adaptive, the adaptive relevance of the later-arising flowering-time-associated variants is less clear. The youngest and lowest frequency variants are often associated with delayed flowering time (fig. 6). Among these, there were no clear candidate loci belonging to pathways directly connected to flowering time. Instead, we identified candidates involved in more peripheral pathways such as nutrient/metal uptake and regulation (e.g., *NRT1*, *NRT1.8*, *ANR1*, *ZIP5*), and light sensing (e.g., *PAPP2C*, AT4G02480). As these other loci were expected to affect flowering time only indirectly and thus only slightly adjust flowering time (Mouradov et al. 2002; Andrés and Coupland 2012; Sanagi et al. 2021), they may have a role in adaptation to other selective pressures such as the volcanic soil, the near-constant photoperiod, or the higher light intensity. Alternatively, these loci may simply represent segregating weakly deleterious mutations. Our results point to the need for studies that examine patterns generated under more diverse and realistic evolutionary models.

The omnigenic model builds on Fisher's infinitesimal model, proposing that highly polygenic complex trait variation is shaped largely by genes in peripheral rather than in core trait pathways (Boyle et al. 2017). According to this model, the high degree of connectivity in biological networks leads to the situation where most expressed genes are only a few steps from the nearest core gene and thus affect a trait through their network interactions (Boyle et al. 2017). Under this model, association signals from pathways other than the flowering-time pathway would be expected due to the complex network of molecular interactions between the flowering-time pathway and more peripheral pathways. Our results are consistent with the idea that populations far from the adaptive optimum are likely to initially move closer using large-effect variants in core genes. However, as the population moves nearer to the adaptive optimum or reaches it, more peripheral genes could predominate. This would be consistent with the idea that traits evolving at or near the optimum (i.e., evolving under stabilizing selection) may often fit an omnigenic model (Boyle et al. 2017), with the recently arising small effect variants largely representing slightly deleterious mutations that have not yet been removed by selection.

We did not find evidence of association for the *CRY2* V367M variant (in the *EDI-Cvi-0* allele) carried by Cvi-0 that was previously shown to have an effect on flowering time (Alonso-Blanco, El-Assal, et al. 1998; El-Din El-Assal et al. 2001, 2003). Common alleles at this locus have also been connected to flowering time variation in Eurasia (Olsen et al. 2004). The main function of *CRY2* is as a light sensor and variation in this gene have been shown to affect many traits in addition to flowering time, including light and temperature responsiveness (Balasubramanian et al. 2006; Sanchez-Bermejo et al. 2015), chromatin condensation (Tessadori et al. 2007), fruit length, ovule number per fruit, percentage of unfertilized ovules and silique number (Botto et al. 2003; el-Assal et al. 2004; Fournier-Level et al. 2013). Although we recapitulated the previous finding of reduced flowering time in the NILs that carried *EDI-Cvi-0* in a Ler-0 background under our experimental conditions, we detected no significant effect of *CRY2* V367M in the CVI natural population. This discrepancy could be due to an epistatic effect that abrogates the *CRY2* effect on flowering time in the CVI genetic background. As we did not find evidence for an epistatic interaction with other segregating variations in Santo Antão that we identified in GWAS, the most likely scenario may be that genetic interaction with a variant fixed in the ancestor of CVI relative to Ler-0 is responsible for the lack of signal in the natural population. Regardless, the *CRY2* V367M allele is at high frequency (90%) in the natural population of Santo Antão, suggesting that it may have been positively selected. Upon colonization from North Africa, CVI *Arabidopsis* would have experienced a sudden shift in several environmental factors and the *CRY2* V367M variant may have been selected due to its effect on other traits. Further work on the effects of *CRY2* V367M on other traits is needed to fully understand its function and relevance under natural conditions. More generally, this finding demonstrates the importance of exercising caution when extrapolating effects observed in one genetic background to other backgrounds. It further points to the need to select appropriate genetic backgrounds, informed by evolutionary history, when examining gene and allelic functions.

Overall, this study provides an empirical example of how genetic architecture built up over time in two relatively young populations that experienced strong directional selection. In addition, it provides insights into how trait architecture is shaped by the combination of demographic and selective forces in natural populations. These results will serve as a useful case for comparison in future work that investigates genetic architectures across traits, populations, and species.

## Materials and Methods

### Genomic Data and Population Structure Analyses

All genomic data on the Moroccan and CVI natural populations were previously published (European Nucleotide Archive codes PRJEB24044 and PRJEB39079, respectively) (Durvasula et al. 2017; Fulgione et al. 2022). We used SNP and InDel variants called with the genomic analysis toolkit (GATK) pipeline and published in (Fulgione et al. 2022). The neighbor-joining tree was produced using the R package *ape* and principal component analysis was conducted using the flag <–pca> in PLINK v.1.90 (Purcell et al. 2007).

### Experimental Conditions and Germplasm

We propagated plants from two CVI, Santo Antão (*n* = 174) and Fogo (*n* = 129), together with plants from Morocco (*n* = 62), in a custom Bronson growth chamber (Brennan et al. 2014; Fulgione et al. 2022). Before sowing, seeds were stratified in the dark in Petri dishes on water-soaked filter paper for one week at 4 °C, and then sown in 7 × 7 cm pots, containing a standard potting compost mix, supplemented with iron. Four replicates of each accession were sown and organized in a randomized block design. Since flowering time heritability tends to be high, and space within the growth chamber was limited, we opted to include a limited number of replicates and instead maximize the number of accessions. Further, compared to a study design with higher line-replication, this design provides high replication at the level of individual alleles (variants) across the population. The chamber was set to track hourly environmental data (temperature, humidity, photoperiod, and precipitation) from Cape Verde, simulating the growing season. Following the precipitation loggers in the field, water was withheld for 26 days after sowing (Fulgione et al. 2022).

Bolting time, that is the number of days from sowing until the appearance of the differentiated floral bud, was scored per individual and the median across replicates was taken as the phenotype per genotype. The term “flowering time” is used throughout the paper to represent the scored bolting time, as the latter is a proxy for the former. At the end of the experiment, the total number of produced seeds was assessed, as a proxy for fitness.

We also grew two Cvi-0×Ler-0 NILs—LCN1–2.5 and LCN1-2.8—and the two parental lines Cvi-0 and Ler-0 to assess the effect of *CRY2*—an a priori candidate–, under CVI simulated conditions. Each line was propagated in four replicates in a randomized block design. Phenotyping was conducted as indicated above.

### Genome-Wide Association Mapping and Genetic Architecture Inference

Broad-sense heritability (H^2^) was calculated in R using a linear mixed-effect model that accounts for the block design, using the function *lmer()* from the package *lme4* (Bates et al. 2015) and narrow-sense heritability (chip heritability) was calculated in GEMMA using BSLMM (Zhou et al. 2013) (see below). To map flowering time, we conducted GWAS using GEMMA v.0.94 (Zhou and Stephens 2012), with some *a posteriori* modifications (see below). We used SNP and InDel variants called with the GATK pipeline and published in (Fulgione et al. 2022). All input files were generated using VCFtools v.0.1.14 (Danecek et al. 2011) and PLINK v.1.90 (Purcell et al. 2007), and the median bolting time across replicates per genotype was used as the phenotype.

To map the genetic basis of flowering time variation, we used a univariate LMM that accounts for population structure with a centered kinship matrix <-gk 1> in GEMMA v.0.94 (Zhou and Stephens 2012). We used the flag <-lmm 4>, which estimates beta per marker as the effect size of an allele, and used the likelihood ratio test to assess evidence for association. For mapping in Santo Antão with *FRI* K232X as a covariate, the flag <-c> was used with a genotype file coded with 0 and 1 for the two alleles. The same approach was used to obtain effect size estimates per marker for seed number.

To infer trait genetic architecture, we used three methods: BSLMM, Lindley (local) scores, and clumping. First, BSLMM (Zhou et al. 2013) was run in GEMMA (Zhou and Stephens 2012). Markov chain Monte Carlo (MCMC) was run with 10,000,000 sampling steps and 2,500,000 burn-in iterations. Median and 95% confidence interval (CI) for PVE and the number of variants with sparse effects (n.gamma) were calculated across ten runs. Second, to compute Lindley scores, also referred to as “local scores’ (Fariello et al. 2017; Bonhomme et al. 2019), from the LMM output, we used available scripts (from https://forge-dga.jouy.inra.fr/projects/local-score/documents) (Bonhomme et al. 2019). Each significant zone was considered a separate candidate locus. Third, clumping of LMM results based on LD was performed using PLINK v.1.90 (Purcell et al. 2007) with the flags <–clump>, <–clump-p1 0.01>, <–clump-kb 1000>, and <–clump-r2 0.8>. Clumps were formed around central “index variants’ which are significantly associated with flowering time at α = 0.01, and by variants that are within 1 Mbp distance and with *r*^2^ > 0.8 with the index marker. A phenotype prediction model was fitted to the local score-identified variants, in addition to *CRY2* V367M, using the *aov()* function in R.

### Testing for an Adaptive Walk

For all analyses in this section, only SNP markers were used. These were filtered from the initial variant call format (VCF) file using the command <–remove-indels> in VCFtools v.0.1.14 (Danecek et al. 2011). SNPs adjacent to apparent complex structural variants were eliminated from the dataset, as these variant calls were unreliable. As noted in the Results section, since *FLC* 3X is fixed in Fogo, where it is included in analyses, it was added manually.

For each candidate locus identified with the local score approach, we took one representative SNP and recalculated its allele frequency and effect size based on the GEMMA estimates. For allele frequency calculations, Moroccan alleles were polarized relative to the *A. lyrata* outgroup. Within CVI, alleles were polarized as described previously (Fulgione et al. 2022). Estimates were recalculated whenever necessary. Candidate loci, represented by a single SNP, were then annotated using SnpEff (Cingolani et al. 2012). The set of candidate loci was further pruned to eliminate SNPs in LD, with an *r*^2^ > 0.5.

For each CVI candidate locus and for an LD-pruned genome-wide set of loci, we also estimated allele ages. We pruned the genomes in Santo Antão and Fogo using PLINK v.0.19 (Purcell et al. 2007) and the flag <–indep-pairwise 50 10 0.3> to produce a set of representative loci across the genome. Specifically, we iterated through 50-SNP windows 50, shifting the window in 10-SNP steps, and selected from variants in the window with *r*^2^>0.3. Then, we estimated allele ages for all candidate SNPs as well as SNPs in the LD-pruned sets in each CVI population using RELATE (Speidel et al. 2019). Ages and 95% confidence intervals were calculated over 200 MCMC runs using Relate´s *TreeViewSample* script. An important note is that RELATE does not allow missing data, so loci with missing data were removed from the analysis. The mutation rate was corrected for missing data across the entire genome and the recombination map was taken from (Salomé et al. 2011) with a correction to assume an outcrossing rate of 5% in the natural populations, based on previous estimates (Bomblies et al. 2010).

## Supplementary Material

msad185_Supplementary_Data

## Data Availability

All scripts are available at https://github.com/HancockLab/CVI-flowering-time-architecture.
