# Supplementary material for: Genetic Architecture of Flowering Time Differs Between Populations With Contrasting Demographic and Selective Histories
Source: Mol Biol Evol. 2023 Aug 21;40(8):msad185. doi: 10.1093/molbev/msad185 (PMC10461413; doi:10.1093/molbev/msad185)
Supplement: msad185_Supplementary_Data [file msad185_supplementary_data.zip › SupplementaryFigures.pdf]

Supplementary Figures

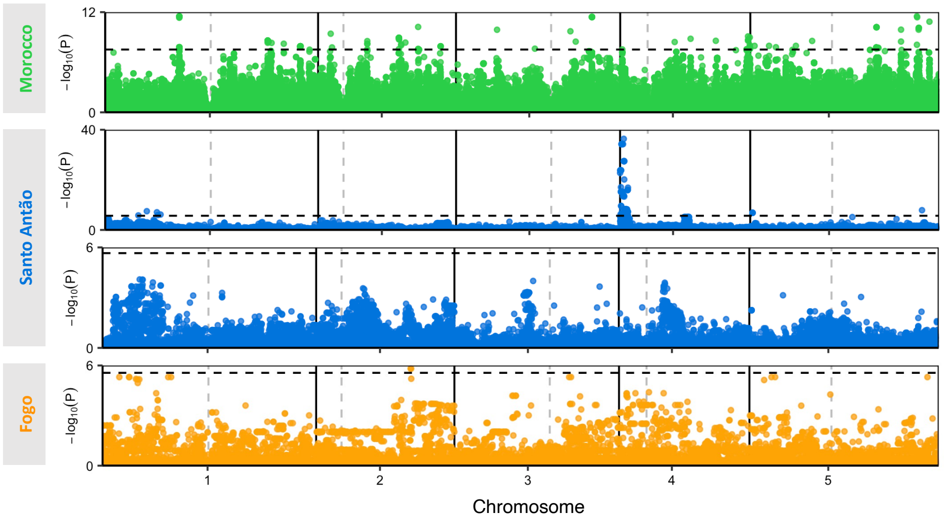

Supplementary Figure 1. GWAS for flowering time. Manhattan plots for flowering time on the three populations from the linear mixed model with Morocco in green, Santo Antão in blue, and Fogo in orange. Mapping in Santo Antão is also shown with *FRI* K232X as a covariate. Genomic position is displayed on the x-axis and significance on the y-axis. The dashed lines represent Bonferroni significance for α=0.05.

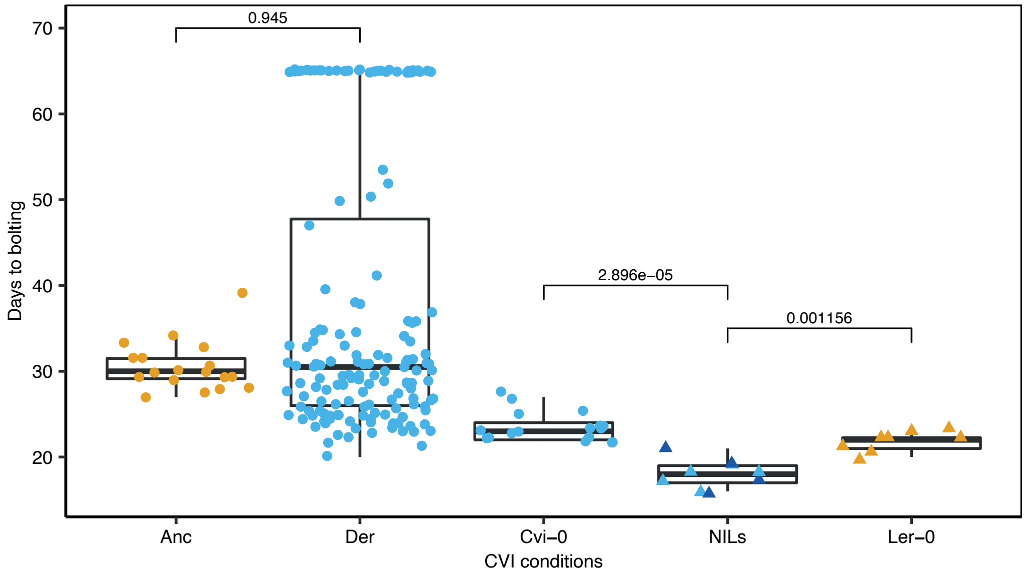

Supplementary Figure 2. *CRY2* V367M effect under CVI simulated conditions. Boxplot of bolting time in days (y-axis) per genotypes (x-axis). ‘Anc’ and ‘Der’ refer to individuals carrying the ancestral and the derived *CRY2* V367M alleles in the natural population of Santo Antão with each dot representing the median across replicates per genotype. ‘Cvi-0’ and ‘Ler-0’ refer to these natural accessions, and ‘NILs’ to the two Cvi-0 x Ler-0 NILs with the EDI locus (each dot represents one replicate and each shade of blue an individual NIL). ‘Anc’ and ‘Ler-0’ represent the effects of the ancestral *CRY2* allele (*CRY2* V367), while Der, Cvi-0 and NILs represent the effects of the derived *CRY2* allele (*CRY2* 367M). Circles represent the CVI genetic background and triangles the Ler-0 genetic background. P-values are shown for the natural population from the LMM (GEMMA), and for the comparison of NILs to the parents using Mann-Whitney U-tests. Boxplots show median (center), 1st and 3rd quartiles (lower and upper bound, respectively) and whiskers represent 95% CI.

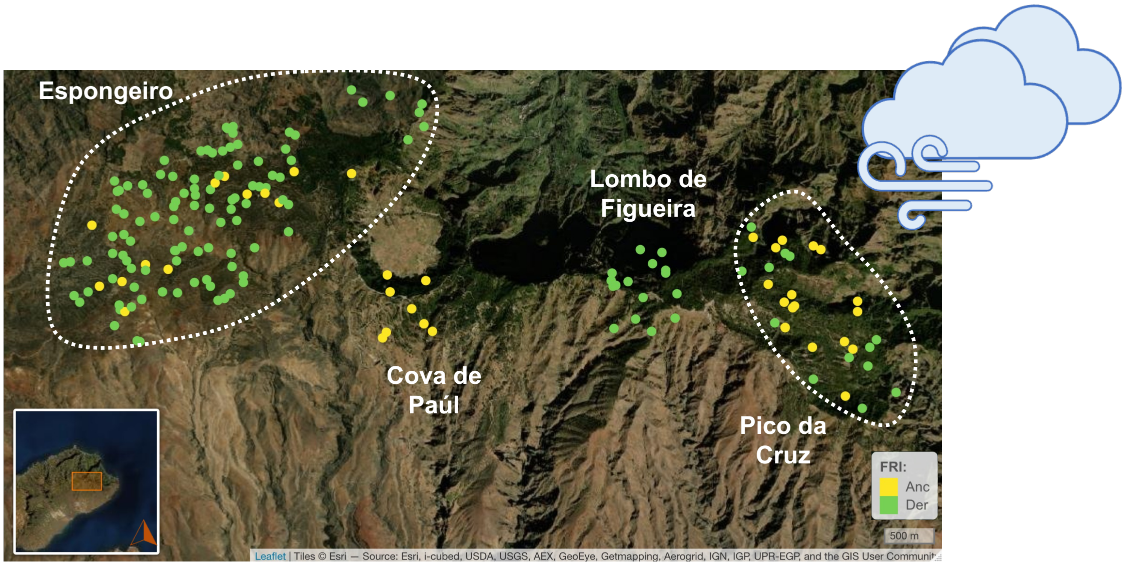

Supplementary Figure 3. Geographical distribution of *FRI* K232X across Santo Antão. Each dot represents one line (accession), colored by the genotype at *FRI*: yellow dots represent individuals carrying the ancestral *FRI* K232 allele, while green dots represent individuals carrying the derived *FRI* 232X allele. Humid northeasterly trade winds are represented by the cloud schematic.
